# Shenfu Injection: A Famous Chinese Prescription That Promotes *HCN4* Activity in Bone Marrow Mesenchymal Stem Cells

**DOI:** 10.1155/2021/9912844

**Published:** 2021-08-17

**Authors:** Xinjun Zhao, Qingmin Chu, Wei Wu, Hui Wu, Song Wang, Lijin Qing, Xiaoxiong Zhou, Zhiyun Luo, Liang Kang, Rong Li

**Affiliations:** Department of Internal Medicine-Cardiovascular, The First Affiliated Hospital of Guangzhou University of Chinese Medicine, Guangzhou 510405, China

## Abstract

We investigated the effects of Shenfu Injection (SFI) on *HCN4* activity in bone marrow mesenchymal stem cells (BMSCs). The sample of BMSCs was divided into six groups: a control group, a high-dose SFI group (0.25 ml/ml), a middle-dose SFI group (0.1 ml/ml), a low-dose SFI group (0.05 ml/ml), an adenovirus-encoded control vector group, and an adenovirus-encoded *HCN4* group. Cell ultrastructure was observed using a transmission electron microscope. Quantitative reverse transcription PCR (RT-qPCR) was performed to detect *HCN4* expression, and *HCN4* activity was detected using the whole-cell patch clamp technique. An enzyme-linked immunosorbent assay was performed to detect cAMP content. Application of flow cytometry confirmed that the isolated cells showed BMSC-like phenotypes. Differentiation of BMSCs in both the SFI and the adenovirus-encoding *HCN4* groups occurred according to the cellular ultrastructure. Application of the whole-cell patch clamp technique revealed that SFI could activate the inward pacing current of BMSCs in a concentration-dependent manner. The RT-qPCR results showed that *HCN4* expression was significantly higher in the high-dose SFI group than in the medium- and low-dose groups, whereas the cAMP content in the overexpressed *HCN4* group decreased significantly; this content in the high-dose SFI group increased significantly. In conclusion, SFI promotes *HCN4* activity in BMSCs, which could explain its treatment effect when administered to patients with cardiovascular diseases.

## 1. Introduction

Sick sinus syndrome (SSS) refers to a group of heart rhythm disorders caused by problems relating to the sinus node. Currently, there is no effective treatment for SSS, and an electronic pacemaker is required to support heart function in SSS patients [1]. However, electronic pacemakers are associated with several defects. For example, external magnetic noise commonly interferes with their functions, leading to complications [2]. In addition, some patients, especially children with congenital sinoatrial node dysfunction, are not suitable subjects for pacemaker insertion [3]. Therefore, the search for new therapeutic strategies for treating cardiovascular diseases has become imperative.

With advancing gene and cell therapy techniques, the potential development of biological pacemakers is a topic of considerable interest [4]. Given their self-renewal and multipotent abilities, stem cells can potentially serve as carriers. Potapova et al. used mesenchymal stem cells as a transplantation platform and transfected genes to establish an in vitro model of a stem cell-based pacing function [5]. Additionally, a previous study demonstrated the *Tbx18*-induced phenotypic transformation of bone marrow mesenchymal stem cells (BMSCs) into pacemaker-like cells [6, 7]. The *HCN4* gene is the molecular basis of pacemaker current, which, through depolarization, provides a large proportion of inward current and plays an important role in the generation and autonomous regulation of the heart rate [8–10]. Moreover, this gene is linked to the diastolic depolarization process of the sinoatrial node [10, 11] and is thought to be a determining channel for cardiocytes [12].

Shenfu Injection (SFI) is a well-known modern Chinese medicinal preparation derived from a traditional formulation known as the “Shenfu Decoction.” This injectable herbal drug is prepared from steamed roots of *Panax ginseng* (red ginseng) and processed lateral roots of *Aconitum carmichaelii* (aconite) [13]. Aconite alkaloids and ginsenosides are thought to be the main active ingredients in Shenfu, and modern pharmacological studies have demonstrated that ginsenosides are the components in Shenfu that promote vasodilation. The alkaloids in Shenfu likely contribute critically to its physiological effects by blocking ion channels [14–16]. In China, SFI has been widely used in the treatment of cardiovascular diseases, such as chronic arrhythmia, coronary heart disease, and acute myocardial infarction [17, 18], whereas the pharmacokinetic properties of SFI have been well investigated [19]; its effects on the pacing function of the sinus node are still not well-understood. In this study, we investigated the effects of SFI on *HCN4* activity in BMSCs. The findings of this study have important implications for the use of SFI in the treatment of heart diseases.

## 2. Materials and Methods

### 2.1. Ethics Statement

The protocol for the use of rabbits in this study was approved by the ethics committee of the First Affiliated Hospital of Guangzhou University of Chinese Medicine (approval number: TCMF1-2019021). Before the surgical procedure, the rabbits were anesthetized with isoflurane. Following the surgery, the animals were euthanized via an intravenous injection of sodium pentobarbitone.

### 2.2. Preparation of BMSCs and Drug Treatments

Under aseptic conditions, bone marrow was harvested from the tibia and femur condyle of one anesthetized male New Zealand rabbit (1 month old; 0.7 kg) as described in a previous study [20]. The bone marrow was repeatedly blown out with a sterile syringe, and the bone marrow cells were fully dispersed within a single cell suspension. Following centrifugation, the cells were suspended in Dulbecco's modified eagle medium (DMEM, Gibco), supplemented with 20% fetal bovine serum (FBS; Hyclone) and cultured at 37°C in an incubator with 5% CO_2_. The surface markers of the BMSCs (CD45, CD29, and CD44) were identified to confirm the phenotype of the isolated cells [21, 22].

The cells were apportioned into six groups for the experiments: a control group, a high-dose SFI group (0.25 ml/ml), a middle-dose SFI group (0.1 ml/ml), a low-dose SFI group (0.05 ml/ml), an adenovirus-encoding control vector group, and an adenovirus-encoding *HCN4* group. SFI was purchased from China Resources Sanjiu Medical and Pharmaceutical Co., Ltd (Batch No. 18100401003). The cells in the SFI groups were treated with SFI for five days, while those in the adenovirus-encoding groups were transfected with the viruses for 48 h before the experiments were performed.

### 2.3. Adenovirus Infection

Adenovirus-encoding *HCN4* or adenovirus-encoding control vector was prepared by the Zhonghong Boyuan Company (Nanchang, Jiangxi Province, China). The *HCN4* sequence (NM_001082707.1) was obtained from NCBI and integrated into the adenovirus following a procedure described in a previous study [23]. BMSCs (2 × 10^5^/well) were seeded into 6-well plates and cultured at 37°C in an incubator with 5% CO_2_. After 24 h, 1 *μ*l of adenovirus (MOI value: 500) encoding *HCN4* was added to the cells in a 5% CO_2_ incubator at 37°C. Monitoring of the fluorescence intensity and cell state was conducted on the second day, and the cells were used in subsequent experiments 48 h after transfection.

### 2.4. Whole-Cell Patch Clamp

After transfection or drug treatments, BMSCs were digested with 0.01% EDTA and 0.25% trypsin. BMSCs were patched using the voltage clamp mode at −70 mV. The *HCN* channel currents in the extracellular fluids of the cells under the voltage clamp were recorded for 3 s at a step hyperpolarization voltage of 10 mV. To avoid a change of ion current caused by differential cell volumes, the current density (PA/PF) on the cell membrane per unit of surface area was calculated according to the membrane capacitance displayed on the Axon200B patch clamp amplifier. The activation curve of the pacemaker ion current of BMSCs was drawn with the pCLAMP software, version 10.7.

### 2.5. Quantitative Reverse Transcription PCR (RT-qPCR)

After transfection or drug treatments, the cell homogenate was collected to extract the total RNA following a procedure described in a previous study [24]. The concentration and purity of RNA (OD260/OD280) were determined using a spectrophotometer. The cDNA was synthesized using an RNA reverse transcription kit (CW2569M, CWBIO, China). The reaction system comprised the following components: dH_2_O, 9.5 *μ*l; cDNA, 1 *μ*l; upstream primer, 1 *μ*l; downstream primer, 1 *μ*l; and 2 × SYBR Green PCR Master Mix, 12.5 *μ*l. The reaction steps were as follows: predenaturation, 95°C for 10 min; denaturation, 95°C for 10 s; annealing, 58°C for 30 s; extending, 72°C for 30 s; 40 cycles. The primer sequences were obtained from General Biosystems Co., Ltd. (Anhui). The relative *HCN4* expression was determined using the 2^−△CT^ method. The following primers were used: HCN4 F 5′-GCTGTCAAAGTGGAGGGAGG-3′, HCN4 R 5′-GCGAGAATTTGTTGACCCCG-3′; GAPDH F 5′-CCACTTTGTGAAGCTCATTTCCT-3′; and GAPDH R 5′-TCGTCCTCCTCTGGTGCTCT-3′.

### 2.6. Enzyme-Linked Immunosorbent Assay

The cAMP level was detected with an enzyme-linked immunosorbent assay (ELISA) kit, following the manufacturer's instructions (MM-020602, Meimian Bio Ltd., Shanghai, China), as described in a previous study [25].

### 2.7. Statistical Analyses

All data were expressed as mean ± standard deviations (SD) and analyzed using the GraphPad Prism 7 software. Significant differences among groups were analyzed by one-way ANOVA followed by a Tukey test (*P* < 0.01).

## 3. Results

### 3.1. AAV-Encoding *HCN4* Promoted *HCN4* Expression in BMSCs

**Figure 1:**
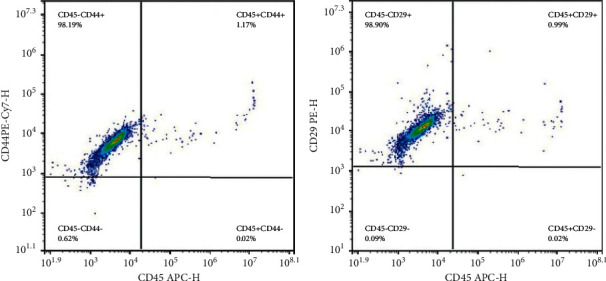
Flow cytometry used to detect the surface markers of bone marrow mesenchymal stem cells (BMSCs). The possession of a BMSC-like phenotype by the isolated cells was confirmed by the positive rates of CD45, CD29, and CD44 (1.19%, 98.9%, and 98.19%, respectively).

Initially, the isolated cells were identified according to the BMSCs' surface markers. As shown in Figure 1, the possession of BMSC-like phenotypes by the isolated cells was confirmed by the positive rates of CD45, CD29, and CD44 (1.19%, 98.9%, and 98.19%, respectively) [21, 22].

**Figure 2:**
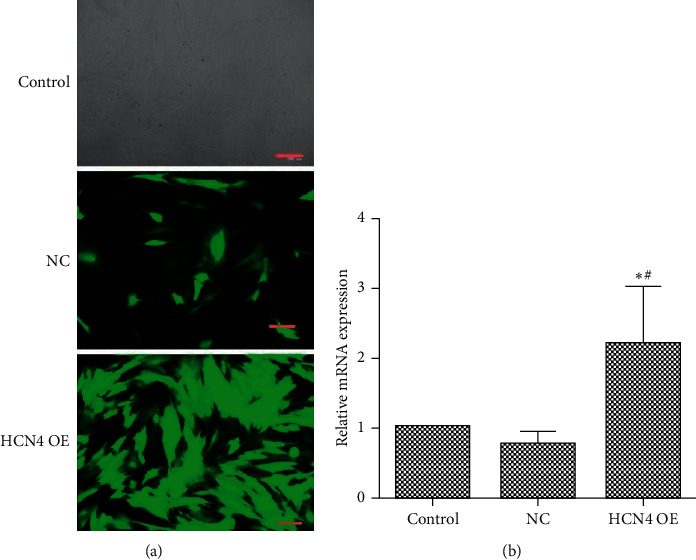
Transfection efficiency of *HCN4*. A representative image of transfection. Green fluorescent protein (GFP) expression was observed under the microscope in both empty and overexpressed groups 48 h after transfection (a). Scale bar: 100 *μ*m. The reverse transcription polymerase chain reaction (RT-PCR) results revealed that *HCN4* expression in the overexpressed group was higher than that in the control group and in the NC group (b), indicating that the transfection was successful. ^*∗*^*P* < 0.05, compared with the control group; ^#^*P* < 0.05, compared with scrambled control (NC) (*N* = 6 in each group).

To assess the effect of SMI on *HCN4* activity, we transfected the BMSCs with AAV-encoding *HCN4* gene treated as a positive control. GFP expression 48 h after transfection was observed under a microscope in both the empty and overexpressed groups (Figure 2(a)). AAV-encoding *HCN4* did not demonstrate cytotoxicity, as evidenced by the healthy growth condition of the cells. The RT-qPCR results showed that *HCN4* expression in the overexpressed group was significantly higher than that in the control and scrambled control groups (Figure 2(b)), indicating that the transfection was successful.

### 3.2. Effects of Shenfu Injection on the Ultrastructure of BMSCs

**Figure 3:**
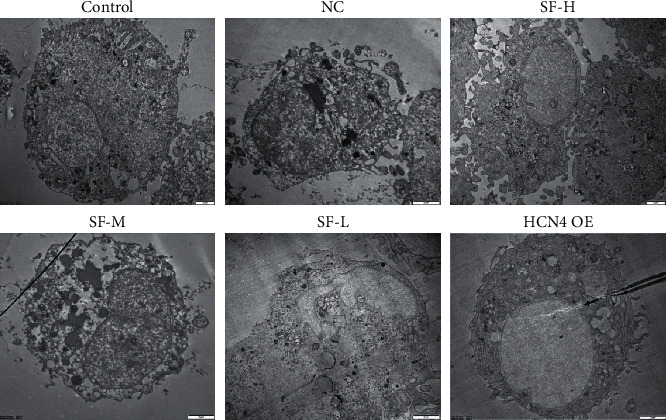
The ultrastructure of bone marrow mesenchymal stem cells (BMSCs) observed with a transmission electron microscope. In the normal saline group, the nuclei of the BMSCs, which had clear nucleoli and nuclear membranes, were positioned on one side. In the Shenfu Injection-treated group, differentiation of BMSCs was conspicuous, evidenced by rough endoplasmic reticulum, increased mitochondria, cell expansion, and increased stromal vesicles. In the overexpressed *HCN4* group, the nuclei were larger, the membrane structure was intact, and there was a clear increase in the rough endoplasmic reticulum, mitochondria, and stromal vesicles compared with those of the control group. Scale bar: 2 *μ*m.

To illustrate the effects of SFI on the ultrastructure of the BMSCs, we examined the cellular ultrastructure of BMSCs using a transmission electron microscope (Figure 3). The nuclei of BMSCs in the normal saline group were located on one side, evidencing clear nucleoli and nuclear membranes. In the SMI-treated groups, BMSCs were clearly differentiated, as evidenced by the rough endoplasmic reticulum, increased mitochondria, cell expansion, and increased stromal vesicles. In the overexpressed *HCN4* group, the nuclei increased in size, while the membrane structure remained intact. Moreover, the rough endoplasmic reticulum, mitochondria, and stromal vesicles were evidently larger than those in the control group. These results indicate that SMI could promote the differentiation of BMSCs.

### 3.3. Effect of Shenfu Injection on the *I*-*V* Curve in BMSCs

**Figure 4:**
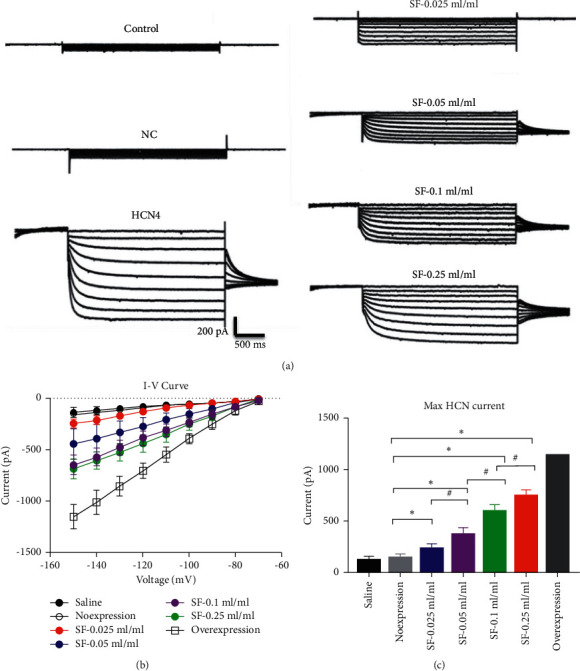
Effect of Shenfu Injection on *I*-*V* curve in bone marrow mesenchymal stem cells (BMSCs). (a) Representative traces; (b) quantified *I*-*V* curve; (c) max *HCN* current. BMSCs in the overexpressed *HCN4* group evidenced hyperpolarization activated inward pacing current, while BMSCs in Shenfu Injection treatment group also showed increased inward pacing current in a concentration-dependent manner (^*∗*^*P* < 0.05; *N* = 10 in each group).

BMSCs could differentiate into cardiac cells with a pacemaking function in vitro. We also detected *HCN4* activity using the patch clamp method. As shown in Figure 4, potassium channel activity was significantly enhanced in BMSCs transfected with AAV-encoding *HCN4*. Overexpression of *HCN4* downregulated the *I/V* curve and increased the max *HCN* current. BMSCs in the SFI treatment group similarly downregulated the *I*/*V* curve and increased the max *HCN* current in a concentration-dependent manner (Figure 4). These findings suggest that SFI promoted *HCN4* activity in BMSCs.

### 3.4. Effect of Shenfu Injection on the Expression of *HCN4* and cAMP in BMSCs

**Figure 5:**
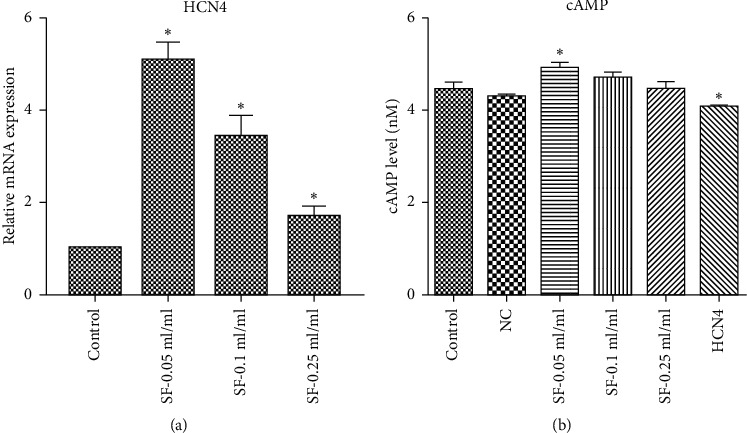
Effect of Shenfu Injection on *HCN4* and cAMP expression in bone marrow mesenchymal stem cells (BMSCs). The expression of *HCN4* at the mRNA level in BMSCs after treatment with Shenfu Injection increased in a concentration-dependent manner (a). The cAMP content was detected by performing an enzyme-linked immunosorbent assay (ELISA) (b). The cAMP content in the high-dose Shenfu Injection group increased, whereas the cAMP content in the overexpressed *HCN4* group decreased significantly. Compared with the control group, ^*∗*^*P* < 0.05 (*N* = 6 in each group).

The effects of SFI on *HCN4* expression at the mRNA level were also evaluated. *HCN4* expression in BMSCs increased significantly after treatment with SFI in a concentration-dependent manner compared with *HCN4* expression in the control group (Figure 5(a)).

Cellular cAMP level was also determined after SFI treatment. Compared with the control group, the cAMP content in the high-dose SFI group increased significantly, and the cAMP content in the overexpressed *HCN4* group decreased significantly (Figure 5(b)). These results suggest that SFI could promote *HCN4* activity, with the likely mechanism being regulation of *HCN4* expression or cAMP levels.

## 4. Discussion

The sinoatrial node, which is located in the right upper atrium, is the heart's natural pacemaker [26]. Symptoms and signs of SSS include abnormal sinus impulses and their transmission, which result from sinus node dysfunction. Clinical symptoms mainly include arrhythmia [27], thromboembolism [28], or any other symptoms requiring pacemaker implantation. Although electronic pacemaker therapy has made great strides, it is still associated with serious flaws [29, 30]. Therefore, biological cardiac pacing has emerged as an important research topic within the field of cardiovascular research.

Mesenchymal stem cells (MSCs) are pluripotent adult stem cells, which are prominent in the bone marrow. The differentiation of MSCs into special cardiomyocytes to treat bradyarrhythmias, such as SSS, is a promising approach [5, 31]. The *HCN* gene is present in all autonomic cells. Like other potassium channels [32], it is associated with hyperpolarization activation, potassium ion permeability, and intracellular cAMP regulation, and it plays a role in the generation of sinus node action potential [33, 34]. *HCN4*, which is sensitive to cAMP, is the main subtype of *HCN* with the largest distribution in the sinoatrial node [35]. The *HCN4* gene is widely used by researchers attempting to construct a biological pacemaker in the field of cardiac biological pacing [33]. Our results showed that the transfection of BMSCs with *HCN4* overexpression adenovirus vector could promote the differentiation of BMSCs and induce the expression of hyperpolarized inward pacing current, which further confirms the important role of *HCN4* in the pacemaker current of sinoatrial node cells.

SFI is widely used in the treatment of various diseases and has cardiovascular protective effects. For example, by regulating apoptosis, it can reduce myocardial dysfunction after resuscitation [36]. Moreover, it has been shown to improve the cardiac function of rats with heart failure [36], stimulate antioxidative effects, and change the level and distribution of phospholipids, with an evident myocardial protective effect on rats with ischemic heart failure [37]. In this study, we treated BMSCs with different doses of SFI. Our results showed that SFI could stimulate differentiation of BMSCs and increase the intensity of inward pacing current. This effect was strengthened with higher concentrations of SFI, but the pacing current intensity remained lower than that of BMSCs transfected with overexpressed *HCN4*. In the high-dose SFI group, the expression and activity of *HCN4* increased along with cAMP content. However, we found that the cAMP content decreased in the overexpressed *HCN4* group. In the absence of cAMP, multiple *HCN4* channel domains and membrane-related intracellular factors in hamster ovary cells reportedly reduce the self-inhibition of *HCN4* channel [38], which indicates that cAMP alone is not sufficient for *HCN4* activation. In this study, decreased cAMP content in the overexpressed *HCN4* group also signaled the activation of cell hyperpolarization which is in a cAMP-independent pathway.

We used SFI to investigate the effect of the Yi Yang Wen Yang method on the biological characteristics of BMSCs as well as “cell biological pacing” and “gene biological pacing.” The findings of this study highlight the function of a traditional Chinese medicine on SSS and provide an experimental and theoretical basis for elucidating the role of traditional Chinese medicine in stem cell differentiation and transplantation in the treatment of major cardiovascular diseases. However, the study had certain limitations. First, in vitro experiments were carried out to investigate the effects of SFI on pacemaker function. In vivo experiments should also be conducted to validate our conclusion. Second, the differentiation of BMSCs should be examined using more indices.

In conclusion, SFI can induce the differentiation of BMSCs and improve the biological function by inducing the expression of the *HCN4* channel gene in BMSCs.

## Data Availability

The datasets used during the present study are available from the corresponding author upon reasonable request.
